# Introduction to flexible nanomaterials: microscopic mechanisms and macroscopic applications

**DOI:** 10.1039/d2na90016e

**Published:** 2022-03-08

**Authors:** Yuan Cheng, Zibiao Li, Junfeng Gao, Hai-Dong Yu, Gang Zhang

**Affiliations:** Monash Suzhou Research Institute, Monash University Suzhou Industrial Park Suzhou 215000 P. R. China; Department of Materials Science and Engineering, Monash University VIC 3800 Australia; Institute of Materials Research and Engineering, A*STAR (Agency for Science, Technology and Research) 2 Fusionopolis Way, Innovis, #08-03 Singapore 138634 Singapore lizb@imre.a-star.edu.sg; Key Laboratory of Materials Modification by Laser, Ion and Electron Beams, Dalian University of Technology, Ministry of Education Dalian 116024 China; Frontiers Science Center for Flexible Electronics, Xi’an Institute of Flexible Electronics (IFE), Northwestern Polytechnical University Xi’an 710072 P. R. China; Institute of High Performance Computing 1 Fusionopolis Way, #16-16 Connexis Singapore 138632 Singapore

## Abstract

Yuan Cheng, Zibiao Li, Junfeng Gao, Hai-Dong Yu and Gang Zhang introduce this themed collection on flexible nanomaterials.
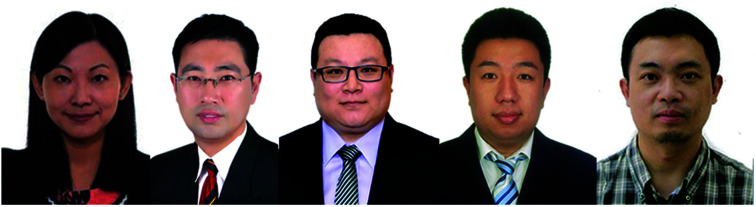

Flexible nanomaterials refer to nano-scaled materials that can be bent and return to their original shape, employed as building blocks for the next generation of electronics, energy, and environmental applications. They can provide an unprecedented flexibility to create a broad range of structures with the combination of polymers, metals and metal derivatives, as well as carbon-based materials. In recent years, scientists and engineers have explored and investigated the physical and chemical properties of flexible nanomaterials for various novel applications. In this themed issue, 13 research articles and reviews are presented in the area of flexible nanomaterials, focusing on the design and synthesis of flexible nanomaterials and polymer composites, applications on electronics, energy harvesting and storage, and environmental remediation.

Two-dimensional (2D) materials are one of the flexible nanomaterials that have attracted significant research interest since the identification of graphene in 2004. Precisely controlled synthesis of 2D materials is critical and of utmost importance. Various top-down and bottom-up approaches are developed to synthesise 2D materials with high quality, such as exfoliation methods, wet chemical approach and chemical vapor deposition (CVD). One contribution, “Continuous orientated growth of scaled single-crystal 2D monolayer films” (DOI: 10.1039/D1NA00545F) reviews two types of strategies, a single seed method and multiple seed technique, of chemical vapor deposition to fabricate 2D materials for various applications. The single-seed method focuses on the ultimate control of density of nucleation into only one nucleus, whereas the multi-seed approach emphasizes on precise engineering of nuclei orientation into uniform alignment. The growth mechanism for the 2D single crystals is presented and their applications in electronics, optics and antioxidation coatings are also discussed.

Electronics as sensors, semiconductors and diodes are constructed using various types of flexible nanomaterials, including nanoribbons, nanosheets and nanodots. The structure and composition of the flexible materials determine the property and performance of the electronics. As reported in “Ligand-assisted deposition of ultra-small Au nanodots on Fe_2_O_3_/reduced graphene oxide for flexible gas sensors” (DOI: 10.1039/D1NA00734C), Au nanodots (NDs) with 1-octadecanethiol (ODT) as the surface ligands are deposited on the surface of α-Fe_2_O_3_/reduced graphene oxide (rGO). The Au ND-ODT modified composite shows enhanced sensing performance due to the fact that Au NDs can facilitate the adsorption of NO_2_ molecules and form ohmic-like contact with rGO. In addition, the surface ligands with less polar terminal groups are beneficial to the charge transfer in the sensing film.

Energy harvesting and storage devices including power generators and batteries with high efficiency are commonly fabricated using flexible nanomaterials. “MC_2_ (M = Y, Zr, Nb, and Mo) monolayers containing C_2_ Dimers: prediction of anode materials for high-performance sodium ion batteries” (DOI: 10.1039/D1NA00422K) predicts the performance of MC_2_ (M = Y, Zr, Nb, and Mo) monolayer containing C_2_-dimers as anode materials for sodium ion batteries using density functional theory computations. The metallic characteristics of the MC_2_ monolayer give rise to excellent electrical conductivity and Na mobility with low activation energies for diffusion. This indicates that the MC_2_ monolayer has great potential to serve as an anode material with a fast charge/discharge rate for sodium ion batteries.

There are many other contributions and applications on flexible nanomaterials that are worthy of attention, including polymer composites, bonding structure and environmental remediation. We would like to thank all the authors and reviewers for their high-quality contributions and support. Special thanks are attributed to the editors of *Nanoscale Advances* for their guidance and support. We do hope the researchers in physics, chemistry, biology and beyond will enjoy reading these articles and be inspired in developing next generation applications with flexible nanomaterials.

## Supplementary Material

